# The responsiveness of the EQ-5D and time trade-off scores in schizophrenia, affective disorders, and alcohol addiction

**DOI:** 10.1186/s12955-015-0315-4

**Published:** 2015-07-31

**Authors:** Michael Sonntag, Hans-Helmut König, Alexander Konnopka

**Affiliations:** Department of Health Economics and Health Services Research, Hamburg Center for Health Economics, University Medical Center Hamburg-Eppendorf, Martinistr. 52, 20246 Hamburg, Germany

**Keywords:** Responsiveness, Schizophrenia, Affective disorders, Alcohol addiction, EQ-5D, TTO

## Abstract

**Objective:**

To compare the responsiveness of the EQ-5D index (German and British tariff), the EQ-5D visual analogue scale (EQ VAS), and time trade-off (TTO) scores in schizophrenia, affective disorders, and alcohol addiction.

**Methods:**

We used a sample of 502 patients and examined the measures at baseline and after 14 months. We used the generic “WHO Quality of Life BREF” (WHOQOL) and the disorder-specific “Global Severity Index” (GSI) as anchors for a relevant improvement in a patient’s health status. In a complete case analysis, we assessed the responsiveness, which is the ability to detect a change given a relevant change on the anchor. We computed the effect sizes (ESs) and standardised response means (SRMs).

**Results:**

In patients with schizophrenia, the ESs and SRMs were large (ES/SRM > 0.8) for the British EQ-5D index (ES_GSI_: 0.93; SRM_GSI_: 0.89; SRM_WHOQOL_: 0.82). In patients with affective disorders, we found large ESs and SRMs for the EQ VAS (ES_GSI_: 1.79; ES_WHOQOL_: 0.90; SRM_GSI_: 1.52; SRM_WHOQOL_: 0.93) and a large ES for the British EQ-5D index (ES_GSI_: 0.88). In patients with alcohol addiction, the ESs and SRMs were large for the EQ VAS (ES_GSI_: 1.40; ES_WHOQOL_: 0.94; SRM_GSI_: 1.04; SRM_WHOQOL_: 0.83). The ESs and SRMs of the German EQ-5D index were consistently lower than those of the British EQ-5D index. Regarding TTO score, ESs and SRMs were generally less than 0.5.

**Conclusions:**

No preference-based instrument was consistently more responsive than others across all mental disorders. While the EQ VAS was the most responsive instrument in patients with affective disorders or alcohol addiction, the British EQ-5D index was reasonably responsive in patients with schizophrenia.

**Electronic supplementary material:**

The online version of this article (doi:10.1186/s12955-015-0315-4) contains supplementary material, which is available to authorized users.

## Introduction

Various approaches and instruments can be used to assess patients’ health related quality of life (HRQOL). In the field of economic evaluation, preference-based instruments are often used to assess HRQOL. These instruments quantify a preference-based valuation of the patient’s current health state by so-called utility weights, which usually range from 0 (death) to 1 (full health) and should be comparable across diseases and disorders. Preference-based instruments can be categorised into direct and indirect instruments [1, 2]. In direct instruments, patients value their own experienced health state or described vignettes directly. Common direct instruments are the time trade-off (TTO) or standard gamble. In indirect instruments, there are two steps to assess utility weights: first, persons of a reference population value a set of predefined generic or condition-specific health states via direct instruments. Based on these valuations, an algorithm is generated to estimate utility weights for each possible health state of the indirect instrument. Second, patients describe their health state on the indirect instrument, and the corresponding utility weight is assigned to the reported health state. Common indirect instruments in patients with mental disorders are the EQ-5D and the SF-6D [3].

Before a preference-based instrument is used in economic evaluations, its psychometric properties should be tested. An important psychometric property is responsiveness, which refers to an instrument’s ability to detect changes in the underlying construct (e.g., health status) over time [4].

Although some studies have already compared the responsiveness of various preference-based instruments [5–10], only a few studies have compared the responsiveness of preference-based instruments in patients with mental disorders [11–14]. In patients with mental disorders, however, no study has compared the responsiveness of direct and indirect preference-based instruments. Thus, the purpose of this study was to compare the responsiveness of the EQ-5D index (British and German tariff), the EQ-5D visual analogue scale (EQ VAS), and the TTO score in patients with schizophrenia, affective disorders, or alcohol addiction.

## Methods

### Subjects and study design

The data of this study came from a study that analysed a new financing model for mental health care in two regions of Northern Germany [15]. The study sample consisted of 170 patients with schizophrenic, schizotypal, or delusional disorders (ICD-10: F2) [16]; 171 patients with affective disorders (ICD-10: F3); and 161 patients with alcohol addiction (ICD-10: F10). The patients were recruited from September 2003 to March 2004 in inpatient, day clinic, or outpatient settings. Five hundred and two patients were assessed at baseline (t0) and after 14 months (t1).

### Instruments

#### EQ-5D

The EQ-5D measures HRQOL by three concepts [17]: (I) the patient-reported “*EQ-5D descriptive system*” has five dimensions (“mobility”, “self-care”, “usual activities”, “pain/discomfort”, and “anxiety/depression”) with three ordinal levels (“no problems”, “moderate problems”, and “severe problems”), resulting in 243 (3^5^) possible health states.

(II) A utility weight (*EQ-5D index*) can be attached to the answers on the EQ-5D descriptive system according to a country-specific tariff. The utility weight is based on the valuation of health states by the general population, which represents the value of a patient’s health state from a general population’s perspective. The EQ-5D index score ranges from health states that are valued worse than death to death (0) to full health (1). The value of the worst possible health state is −0.59 on the British EQ-5D index (EQ-5D index-UK) [18] and −0.21 on the German EQ-5D index (EQ-5D index-G) [19]. Despite the analysis being of a German patient sample, we used both EQ-5D indexes because the estimation of the EQ-5D index-G was based on a rather small sample (n_German_ = 334 vs. n_UK_ = 2997) and on the valuation of fewer health states (36 vs. 43). Because most of the valued health states had large standard deviations in relation to the mean in the German sample, a regression model without non-significant variables was computed. Based on the study’s results, the EQ-5D index-G score does not change if the patients have improved from level 2 (“moderate problems”) to level 1 (“no problems”) in the dimension “anxiety/depression”. Thus, the EQ-5D index-G scores must be considered as less precise.

(III) Respondents rate their current health state on the *EQ VAS*, which is a rating scale similar to a thermometer that ranges from 0 (worst imaginable health state) to 100 (best imaginable health state). The EQ VAS represents the value of HRQOL from the respondent’s perspective. Based on economic theory, the EQ VAS does not represent choice-based preferences and thus produces no utility weights [20–22]. However, the EQ VAS has been shown to explain a substantial proportion of the variance in standard gamble and TTO [23]. Despite these theoretical limitations, we labelled the EQ VAS as a preference-based instrument. To better compare the preference-based instruments, we divided the EQ VAS score by 100.

#### TTO

The TTO assessment was based as closely as possible on the protocol of the Measurement and Valuation of Health (MVH) Group [24] (see TTO study protocol in the Additional file 1). Accordingly, patients had to choose between two hypothetical alternatives in personal interviews: in the first alternative, patients stayed in their current health state for 10 years followed by death; in the second alternative, patients stayed x years at full health followed by death (restricted to x ≤ 10 years). Time *x* was varied until the patient was indifferent between the two alternatives, leading to a utility weight of “x/10”. If the patient preferred zero years at full health (which equals immediate death) to 10 years in the current health state, the patient valued their current health state worse than death. In this case, utility weights can be negatively infinite for health states valued worse than death.

#### WHOQOL-BREF

The WHOQOL-BREF is a self-administered generic HRQOL measure referring to the previous two weeks. It is a short version of the WHOQOL-100 measure and contains 26 items, each rated on a 5-point Likert scale. Two items assess the patients’ overall perception of quality of life and their health, respectively. The other 24 items can be summarised in a global score. The global score ranges from 0 (worst) to 100 (best). It has been shown that the WHOQOL-BREF is valid, reliable, and responsive in patients with schizophrenia, affective disorders, or alcohol addiction [25–27].

#### SCL-90R

The SCL-90R is a self-administered questionnaire measuring the distress induced by mental symptoms in the previous 7 days [28]. Each of the 90 items ranges from 0 (best) to 4 (worst). The items can be aggregated to the Global Severity Index (GSI), which represents the mean of all the item scores ranging from 0 (best) to 4 (worst).

### Analysis

We only analysed the responsiveness in patients with an improved health status because the number of patients with a deteriorated health status was very small. We conducted a complete case analysis regarding the preference-based instruments. We used both the GSI and WHOQOL-BREF global scores as anchors to identify patients with an improved health status. Because of the lack of available minimal important differences in the anchors, a relevant health status change on the anchor was defined as more than ±0.5 standard deviations (baseline) for each mental disorder [29, 30].

In regard to the TTO, 15 patients had utility weights less than −1.00 (lowest TTO score: −19) at baseline. Because outliers strongly influence the coefficients of the ES and SRM in small sample sizes, we censored the range of the TTO from −1.00 to 1.00.

We assessed the correlation between the preference-based instruments and each anchor to ensure that the constructs of both instruments were similar. We computed Spearman’s rank correlation coefficient because the preference-based instruments did not follow a normal distribution. According to Cohen, we defined a correlation coefficient as small if 0.1 ≤ |r_s_| < 0.3, moderate if 0.3 ≤ |r_s_| < 0.5, and large if |r_s_| ≥ 0.5 [31].

Responsiveness can be assessed in various ways [32–35]. In our analysis, we computed effect sizes (ESs, mean of change scores divided by the standard deviation at baseline) and standardised response means (SRMs, mean of change scores divided by the standard deviation of the change scores) of each preference-based instrument given a relevant improvement on the anchor. Both responsiveness scores provide information on the magnitude of change in relation to the level of variation at baseline (ES) or in relation to the level of variation in change scores (SRM). Thus, the interpretation of responsiveness differs between ES and SRM according to how the level of variation is defined. According to Cohen [31], we defined the scores of ES and SRM as trivial from ≥ |0.1| to < |0.2|, as small from ≥ |0.2| to < |0.5|, as medium from ≥ |0.5| to < |0.8|, and as large if ≥ |0.8|.

The statistical analyses were conducted using the Statistical Package for the Social Sciences (version 18, SPSS Inc., Chicago, IL, USA).

## Results

### Patient characteristics

The patient characteristics at baseline are shown in Table 1. Most of the schizophrenic patients were male (57.6 %), unmarried (61.2 %), and lived alone (33.7 %). The patients with affective disorders were mostly female (69.0 %), married (37.4 %), and lived with their spouse/partner (48.8 %). Most of the patients with alcohol addiction were male (71.4 %), separated/divorced (36.6 %), and lived alone (50.9 %). Across all disorders, most patients had a lower secondary school degree.

**Table 1:** Patient characteristics at baseline

Characteristics/ diagnosis	Patients with schizophrenia	Patients with affective disorders	Patients with alcohol addiction
N	170	171	161
Gender: n (%)			
Male	98 (*57.6*)	53 (*31.0*)	115 (*71.4*)
Female	72 (*42.4*)	118 (*69.0*)	46 (*28.6*)
Family status: n (%)			
Unmarried	104 (*61.2*)	49 (*28.7*)	42 (*26.1*)
Married	31 (*18.2*)	64 (*37.4*)	47 (*29.2*)
Separated/divorced	29 (*17.1*)	38 (*22.2*)	59 (*36.6*)
Widowed	6 (*3.5*)	20 (*11.7*)	13 (*8.1*)
Living situation: n (%)			
Alone	57 (*33.7*)	65 (*38.3*)	82 (*50.9*)
With spouse/partner	41 (*24.3*)	83 (*48.8*)	68 (*42.2*)
Nursing home	42 (*24.8*)	7 (*4.1*)	4 (*2.5*)
Other forms	29 (*17.2*)	15 (*8.8*)	7 (*4.4*)
Education: n (%)			
Low	79 (*46.4*)	79 (*46.5*)	91 (*56.9*)
Middle	53 (*31.2*)	61 (*35.9*)	47 (*29.4*)
High	36 (*21.2*)	29 (*17.1*)	18 (*11.2*)
Other	2 (*1.2*)	1 (*0.5*)	4 (*2.5*)
Age: mean (SD)	41.0 (*10.9*)	48.3 (*15.5*)	47.8 (*11.0*)

Italics: percentage of the item; SD: standard deviation

### Score distribution at baseline

#### Scores of instruments used as anchors

The patients with schizophrenia showed the highest mean WHOQOL-BREF score (57.4) and the lowest mean GSI score (0.62; Table 2). The patients with affective disorders reported the lowest mean WHOQOL-BREF score (44.8) and the highest mean GSI score (0.95). In the patients with alcohol addiction, the mean WHOQOL-BREF score was 49.4 (SD: 21.7) and the mean GSI score was 0.65 (SD: 0.60), indicating moderate psychopathological problems.

**Table 2:** Descriptive statistics of the EQ-5D, time trade-off score, WHOQOL-BREF, and GSI at baseline

	Possible range of score (worst-best)	N (schizophrenia/ affective disorders/alcohol addiction)^a^	Schizophrenia		Affective disorders		Alcohol addiction		German population norms^b^		
Instruments			Mean (SD)	Range	Mean (SD)	Range	Mean (SD)	Range	N	Mean (SD)	Range
EQ-5D index-G	−0.21 – 1.00	106/101/72	.855 (.255)	0.11 – 1.00	.828 (.214)	0.11 – 1.00	.840 (.200)	0.03 – 1.00	3552	.938 (.126)	−0.20 – 1.00
EQ-5D index-UK	−0.59 – 1.00		.735 (.278)	−0.18 – 1.00	.678 (.266)	−0.18 – 1.00	.710 (.230)	−0.15 – 1.00	3552	.908 (.166)	−0.59 – 1.00
EQ VAS score	(0 – 100) /100		.691 (.182)	0.2 – 1.00	.582 (.242)	0 – 1.00	.583 (.212)	0.02 – 1.00	3546	.774 (.193)	0 – 1.00
Time trade off	−1.00 – 1.00		.810 (.310)	−1.00 – 1.00	.700 (.380)	−0.67 – 1.00	.650 (.370)	−0.54 – 1.00	n.a.		
WHOQOL-BREF	0 – 100	170/171/161	57.4 (21.0)	0 – 100	44.8 (24.1)	0 – 100	49.4 (21.7)	0 – 100	n.a.		
GSI	4 – 0	146/140/147	0.62 (0.52)	2.64 – 0	0.95 (0.71)	3.17 – 0	0.65 (0.60)	3.52 – 0	n.a.		

^a^ Number of observations varied due to missing values; ^b^ data based on [42]; EQ-5D index-G/UK: German/British EQ-5D index; GSI: global severity index; n.a.: not available for the German population; SD: standard deviation; VAS: visual analogue scale

#### Scores of preference-based instruments

Across all disorders, the mean EQ-5D index-G score was the highest, followed by the TTO, EQ-5D index-UK, and EQ VAS scores, except in the patients with alcohol addiction for whom the mean EQ-5D index-UK score (0.710) was higher than the mean TTO score (0.650, Table 2). No preference-based instrument showed floor effects at baseline (results not shown). Both EQ-5D indexes and TTO showed ceiling effects in contrast to the EQ VAS (<5 %). In the patients with schizophrenia, 25 % (measured by the EQ-5D index-UK), 43 % (EQ-5D index-G), and 45 % (TTO) of all patients reported full health. In the patients with affective disorders, 15 % (EQ-5D index-UK) and 34 % (EQ-5D index-G/TTO) of all patients reported full health. In the patients with alcohol addiction, 10 % (EQ-5D index-UK), 31 % (EQ-5D index-G), and 26 % (TTO) of all patients reported full health.

Comparing the means of the patient population with those of the German population norms, the available means of the German population norms were consistently higher than the means of each patient group.

### Correlation between scores of the preference-based instruments and scores of the anchors

Across all disorders, we mostly found moderate Spearman’s rank correlation coefficients between the EQ-5D index-G/TTO scores and the WHOQOL-BREF/GSI scores. The EQ-5D index-UK scores and the EQ VAS scores had both moderate and large correlations with the scores of both anchors (Table 3).

**Table 3:** Correlation between the EQ-5D scores, the time trade-off scores and the scores of the anchors at baseline

			Spearman rank correlation coefficient			
*Anchor*	*Disorder*	*n*	*EQ-G*	*EQ-UK*	*EQ VAS*	*TTO*
WHOQOL-BREF	F1	63	.234*	.339*	.635*	.390*
	F2	106	.469*	.570*	.472*	.194*
	F3	101	.381*	.495*	.758*	.500*
GSI	F1	72	-.171	-.345*	-.356*	-.345*
	F2	88	-.622*	-.744*	-.469*	-.281*
	F3	82	-.465*	-.649*	-.736*	-.397*

* *p* ≤ 0.05

EQ-G/UK: German/British EQ-5D index; F1: patients with alcohol addiction; F2: patients with schizophrenia; F3: patients with affective disorders; GSI: global severity index; TTO: time trade-off; VAS: visual analogue scale

### Responsiveness

#### British EQ-5D index

In the patients with schizophrenia, there were large ESs and SRMs on the EQ-5D index-UK (ES_GSI_: 0.93; SRM_GSI_: 0.89; SRM_WHOQOL-BREF_: 0.82). In the patients with affective disorders, there was a large ES (ES_GSI_: 0.88) anchored by the GSI (Table 4). In the patients with alcohol addiction, the ESs and SRMs that were anchored by the WHOQOL-BREF were small, whereas those anchored by the GSI were medium (ES_GSI_: 0.64; SRM_GSI_: 0.56).

**Table 4:** Responsiveness of the German and British EQ-5D index, EQ VAS, and time trade-off scores for patients with improved health status

		Mean of change scores (SD baseline)					Effect size				Standardised response mean			
*Anchor*	*Disorder*	*n*	*EQ-G*	*EQ-UK*	*EQ VAS*	*TTO*	*EQ-G*	*EQ-UK*	*EQ VAS*	*TTO*	*EQ-G*	*EQ-UK*	*EQ VAS*	*TTO*
WHO QOL-BREF	*F1*	*46*	*.02 (.20)*	*.06 (.23)*	*.18 (.19)*	*.10 (.38)*			**0.94**				**0.83**	
	*F2*	*46*	*.16 (.30)*	*.23 (.34)*	*.11 (.18)*	*.09 (.39)*	0.54	0.68	0.61		0.63	**0.82**	0.70	
	*F3*	*54*	*.05 (.20)*	*.12 (.25)*	*.19 (.23)*	*.13 (.42)*			**0.90**			0.51	**0.93**	
GSI	*F1*	*20*	*.09 (.22)*	*.17 (.26)*	*.26 (.19)*	*.11 (.32)*		0.64	**1.40**			0.56	**1.04**	
	*F2*	*17*	*.20 (.29)*	*.28 (.30)*	*.10 (.18)*	*.02 (.48)*	0.66	**0.93**	0.58		0.63	**0.89**	0.63	
	*F3*	*21*	*.11(.22)*	*.22 (.25)*	*.29 (.16)*	*.25 (.45)*	0.50	**0.88**	**1.79**	0.56		0.73	**1.52**	0.74

For clarity, we displayed only effect sizes (ES) and standardised response means (SRM) ≥ |0.5|. Large ESs and SRMs (ES/SRM ≥ |0.8|) were printed bold

EQ-G/UK: German/British EQ-5D index; F1: patients with alcohol addiction; F2: patients with schizophrenia; F3: patients with affective disorders; GSI: global severity index; SD: standard deviation; TTO: time trade-off; VAS: visual analogue scale

#### German EQ-5D index

In the patients with schizophrenia, the ESs and SRMs on the EQ-5D index-G were medium (ES_WHOQOL-BREF_: 0.54; ES_GSI_: 0.66; SRM_WHOQOL-BREF_: 0.63; SRM_GSI_: 0.63). In the patients with affective disorders or alcohol addiction, the ESs and SRMs were generally small.

#### EQ VAS

In the patients with schizophrenia, we found medium ESs and SRMs (ES_WHOQOL-BREF_: 0.61; ES_GSI_: 0.58; SRM_WHOQOL-BREF_: 0.70; SRM_GSI_: 0.63) on the EQ VAS. In the patients with affective disorders, we found large ESs and SRMs (ES_WHOQOL-BREF_: 0.90; ES_GSI_: 1.79; SRM_WHOQOL-BREF_: 0.93; SRM_GSI_: 1.52). In the patients with alcohol addiction, the ESs and SRMs were large (ES_WHOQOL-BREF_: 0.94; ES_GSI_: 1.40; SRM_WHOQOL-BREF_: 0.83; SRM_GSI_: 1.04).

#### TTO

In the patients with schizophrenia, we found small and trivial ESs and SRMs on the TTO score. In the patients with affective disorders, we found a medium ES and SRM (ES_GSI_: 0.56; SRM_GSI_: 0.74) anchored by the GSI. In the patients with alcohol addiction, the ESs and SRMs were small or trivial.

## Discussion

In this study, we analysed the responsiveness of the EQ-5D index-UK, the EQ-5D index-G, the EQ VAS, and the TTO score in patients with schizophrenia, affective disorders, or alcohol addiction with an improved health status according to the WHOQOL-BREF or GSI, which were used as anchors. We computed the ES and SRM to assess and compare the responsiveness of the four preference-based instruments.

The correlation coefficients between the preference-based instruments and the anchors were mainly moderate and large, indicating that the preference-based instruments captured relevant aspects of HRQOL that were covered in the anchors. Thus, the constructs of the preference-based instruments were similar to the constructs of both anchors.

Using two anchors, in total, we computed 24 ESs/SRMs (4 preference-based instruments *2 anchors *3 mental disorders). Of the 24 computed ESs/SRMs, we found six large ESs and six large SRMs. With the large ESs, the corresponding SRM was large in five comparisons and medium in one comparison and vice versa. Thus, the level of responsiveness between ES and SRM was consistent in five of the six comparisons, indicating that both responsiveness statistics may lead to the same level of responsiveness. However, this evidence does not imply that only one of both responsiveness statistics is sufficient to assess the level of responsiveness. Various factors influence the level of responsiveness in each method. Whereas the distribution of the baseline scores influences the ES, the SRM is influenced by the distribution of change scores (particularly the change scores of outliers) [35, 36]. Depending on the particular study population, ES and SRM can differ according to their level of responsiveness.

In patients with affective disorders or alcohol addiction, the EQ VAS appeared to be the most responsive instrument. This may be because of the different characteristics of the EQ VAS and the EQ-5D index. Compared to the EQ-5D index, the EQ VAS covers all dimensions that the patients feel are subjectively important in their HRQOL. In contrast, the EQ-5D descriptive system predefines the HRQOL dimensions that are externally considered important for patients’ HRQOL. Additionally, the EQ-5D descriptive system only differentiates between three levels of severity. Patients may be reluctant to respond to an improvement in the corresponding EQ-5D dimensions because the improvements may be considered marginal from the patient perspective. In the EQ VAS, however, patients can respond to these marginally perceived improvements with small change scores. Therefore, the EQ VAS may encompass more subjectively important HRQOL dimensions than the EQ-5D index [37] and may even detect marginally perceived improvements in patients’ HRQOL.

In patients with schizophrenia, the EQ-5D index-UK appeared to be the most responsive instrument with large ESs and SRMs despite large ceiling effects at baseline. The EQ VAS had a lower level of responsiveness, which contrasts our previous argument that the EQ VAS encompasses more subjectively important HRQOL dimensions than the EQ-5D index. A reason may be that the descriptive system of the EQ-5D covers most of the relevant HRQOL dimensions in this patient group. The mean change score of the EQ-5D index-UK was higher than those of the other preference-based instruments. However, our results should be interpreted with caution because previous studies showed that the responsiveness of the (British and German) EQ-5D index was low in larger patient samples [13, 14, 38]. There may be two reasons for the differences in the level of responsiveness. The first reason may be our smaller sample size. The second reason may be that the assessment of responsiveness differed from our study. Whereas Mulhern et al. [14] assessed the responsiveness with SRM based on all patients and no anchor, McCrone et al. [13] used the SRM in relevantly improved patients based on a disorder-specific anchor. Konnopka et al. [38] assessed the responsiveness with the receiver operating characteristic curve using other anchors (EQ-5D transition question and a schizophrenia-specific measure). However, it is difficult to decide whether an anchor is required at all and if so, which anchor may be the most suitable (“gold standard”) for the assessment of responsiveness (transition question, disease-specific, or generic anchor). When using no anchor, the level of responsiveness is highly depended on the particular treatment effects and is based on “statistically significant” change, which may not necessarily constitute a meaningful change in a patient’s health status [32]. However, the number of patients which can be used for the analysis is larger than that if using anchor-based methods. In using an anchor, the change in the preference-based instrument can be linked to a meaningful change in the anchor. When using transition questions, there is a definite indicator for a change. However, if the transition question has various levels (e.g., slightly, a little, a lot), it is unclear which level of the change is meaningful for patients. Although disease-specific anchors can detect marginal clinical changes in a patient’s health status, the question is whether each marginal clinical change leads to a change in the preference-based instrument. When using a generic anchor, it is debateable whether the generic anchor is sensitive enough to detect meaningful health status changes in the disease. Additionally, the assessment of an anchor’s minimal important difference (MID) is influenced by the choice of the sample and the MID method leading to different MIDs of the same anchor [39, 40].

Both EQ-5D indexes are based on the same descriptive system. In contrast to the EQ-5D index-UK, which had two large ESs and SRMs, the EQ-5D index-G had no large ESs or SRMs. The lower responsiveness of the EQ-5D index-G may be a result of the instrument’s insensitivity to a change from level 2 to level 1 in the EQ-5D dimension “anxiety/depression”. It is expected that patients with mental disorders report the most changes in this EQ-5D dimension.

To estimate the EQ-5D index scores, the TTO method was used to value predefined health states of the EQ-5D descriptive system. Thus, one may expect that the TTO score and the EQ-5D index have a similar level of responsiveness. In our study, we found two large and two medium ESs and two large and three medium SRMs in the EQ-5D index-UK. The TTO score, however, had only one medium ES and one medium SRM. This inconsistent level of responsiveness between the TTO score and the EQ-5D index-UK may be based on two major reasons. First, the TTO scores of our study referred to the valuation of the patients’ own experienced and unrelated health state, whereas the TTO scores used for the EQ-5D index referred to the valuation of predefined and hypothetical health states. Thus, the construct of the valued health state is different between the TTO of our study and the TTO used for the EQ-5D index. Second, the TTO task may be more challenging for patients than describing their current health state in the EQ-5D descriptive system.

### Studies comparing the responsiveness of preference-based instruments in patients with mental disorders

We did not find any studies comparing the preference-based instruments used in this study. However, four studies compared the responsiveness of the EQ-5D index-UK and the SF-6D in patients with mental disorders. Gerhards and colleagues [11] compared the responsiveness of the SF-6D and the EQ-5D index-UK in patients with depression using the ES and SRM. The ESs and SRMs ranged from small to large and were anchored by a disease-specific instrument and a patient self-reported global rating of change. The SF-6D had slightly higher ESs and SRMs than the EQ-5D index-UK. The authors concluded that both instruments can be applied in assessing health effects in patients with depression.

Lamers and colleagues [12] assessed the responsiveness of the SF-6D and the EQ-5D index-UK in patients with mood and/or anxiety disorders using the SRM. Without using an anchor, the SRM of the SF-6D was consistently higher (SRM ≈ 0.83) than the SRM of the EQ-5D index-UK (SRM ≈ 0.46) in each severity subgroup.

McCrone and colleagues [13] compared the responsiveness of the EQ-5D index-UK with the SF-6D in patients with schizophrenia using the SRM. With an improvement of the patients’ health status anchored by a disease-specific instrument, the SRM of the EQ-5D index-UK and the SF-6D were identical but small (SRM = 0.39).

Mulhern and colleagues [14] assessed the responsiveness of the EQ-5D index-UK and the SF-6D in patients with schizophrenia using the SRM. They included all patients who had completed both instruments at both time points. Without referring to an anchor, the SRM of the EQ-5D index-UK and the SF-6D were identical but trivial (SRM = 0.12). In our study, however, we found that the EQ-5D index-UK was reasonably responsive, irrespective of the responsiveness statistic.

### Strengths and limitations

This study was the first to compare the responsiveness of the EQ-5D index-UK, the EQ-5D index-G, the EQ VAS, and the TTO score in patients with schizophrenia, affective disorders, or alcohol addiction. Additionally, we used the ES and the SRM to provide more insight into a potential convergence or divergence in the level of responsiveness by applying these two different responsiveness statistics.

However, the number of patients with each mental disorder may have been too small to draw general conclusions about whether the preference-based instrument is responsive in each mental disorder. Nonetheless, we could identify some hints about which preference-based instrument was more responsive than the others in each mental disorder using the same patients. Another limitation was the use of 0.5 standard deviations as the definition of a relevant change in the anchor. However, we did not find a valid definition of a relevant change in both anchors. Finally, we used generic anchors instead of disease-specific measures to conduct the comparisons of responsiveness across the mental disorders. Therefore, additional comparisons of preference-based instruments against other external instruments may be required.

### Implications for future research

Because many preference-based instruments are available in the literature, the assessment and comparison of the responsiveness of these instruments should be extended to other mental disorders and diseases. Particularly, researchers should compare the responsiveness between generic (such as the EQ-5D) and disorder-specific preference-based instruments (e.g., the DEMQOL-U for patients with dementia [41]). Disorder-specific preference-based instruments may encompass more specific dimensions that patients feel are relevant for the valuation of their health status. Moreover, researchers may focus on the assessment of responsiveness in patients with a deteriorated health state.

## Conclusion

No preference-based instrument was consistently more responsive than the others across all mental disorders. In the patients with schizophrenia, the EQ-5D index-UK appeared to detect relevant changes in contrast to other studies. In the patients with affective disorders or alcohol addiction, the EQ VAS appeared to be the most responsive instrument. The responsiveness of the EQ-5D index-G and the TTO score was low in each mental disorder. More responsiveness studies comparing various preference-based instruments are required.

## Additional file

Additional file 1:
**Time trade-off study protocol based on the checklist of Attema et al. [** 43 **]**. (DOC 61 kb)

## Abbreviations

ES: Effect size
GSI: Global severity index
HRQOL: Health related quality of life
SRM: Standardised response mean
TTO: Time trade-off
WHOQOL: World Health Organisation quality of life
VAS: Visual analogue scale
