# Supplementary material for: The responsiveness of the EQ-5D and time trade-off scores in schizophrenia, affective disorders, and alcohol addiction
Source: Health Qual Life Outcomes. 2015 Jul 31;13:114. doi: 10.1186/s12955-015-0315-4 (PMC4632463; doi:10.1186/s12955-015-0315-4)
Supplement: Additional file 1: — Time trade-off study protocol based on the checklist of Attema et al. [ 43 ]. (DOC 61 kb) [file 12955_2015_315_MOESM1_ESM.doc]

Additional file 1: Time trade-off study protocol based on the checklist of Attema et al.

| *Methodological question* | Specification |
| --- | --- |
| 1. What value range was assessed? | Both states BTD and WTD |
| 1. What method was used for valuation of worse than dead states? | MVH protocol |
| 1. What was the disease duration? | 10 years |
| 1. Was the smallest tradable unit listed? | yes for the interviewer (half year in current health state followed by 9.5 years in full health) |
| 1. Was the lead or lag time listed? | yes for the interviewer |
| 1. What iteration procedure was used? | MVH fixed sequence |
| 1. What was the response scale? | Years lived in full health |
| *Procedural question (direct valuation used)* | |
| 1. What was the mode of administration? | Face-to-face interviews |
| 1. Were visual aids used? | Yes, TTO board |
| 1. What context effects were considered? | Not done |
| 1. What was the sampling frame? | Patients |
| 1. Were all health state values observed? | Only own health valued |
| 1. How was the health state described? | Own health |
| 1. How were the health state descriptions generated? | Full health defined as no problems on any dimension of the EQ-5D |
| 1. How was the text of the description structured? | Narrated without label |
| *Analytical question* |  |
| 1. What exclusion criteria were used? | Trade-off, non-traders/ logical errors |
| 1. How was the best possible health defined? | Perfect health |
| 1. How were WTD values analysed? | Transformation *x*/(1-*x*) |
| 1. Was the TTO adjusted for time-preference? | No |

BTD: better than death; MVH: measurement and valuation of health; TTO: Time trade-off; WTD: worse than health.
